# Refractory right ventricular myocarditis induced by immune checkpoint inhibitor despite therapy cessation and immune suppression

**DOI:** 10.1186/s40959-023-00165-2

**Published:** 2023-03-20

**Authors:** Khan O. Mohammad, Hanna Fanous, Sneha Vakamudi, Yan Liu

**Affiliations:** Department of Internal Medicine, Dell Medical School at The University of Texas, Austin, TX USA

**Keywords:** Immune check point inhibitor, Right ventricle, Myocarditis, Abatacept, CTLA-4

## Abstract

**Background:**

Immune checkpoint inhibitors (ICIs) are currently widely used for treatment of various types of cancers. ICI-induced myocarditis, though uncommon, accounts for high risk of major adverse cardiac events and mortality, which makes appropriate diagnosis important. We here present a unique, challenging case of ICI-induced, refractory and isolated right ventricular (RV) myocarditis.

**Case presentation:**

A 32-year-old female with breast cancer presented with newly onset chest pain and dyspnea shortly after initiation of Pembrolizumab. Coronary angiography showed normal coronary arteries and a cardiac magnetic resonance (CMR) revealed myocarditis involving the right ventricle with chamber dilation and severe dysfunction. ICI therapy was stopped, and high dose steroid therapy was initiated and symptoms resolved. However, three months after initial presentation, the patient was hospitalized for DKA and decompensated right heart failure, and a repeat cardiac MRI at that time showed recurrent, isolated right ventricular myocardial inflammation/edema without LV involvement. High dose steroid therapy was started again and at 6-month follow up, surveillance CMR continued to show persistent right-sided myocarditis, patient was eventually treated with Abatacept with resolution of HF symptoms, RV dysfunction and biomarkers at 10-month follow up.

**Conclusions:**

We describe a unique case of isolated ICI-induced right ventricular myocarditis leading to right ventricular failure, that was refractory despite ICI therapy cessation and immune suppression by repeated high dose steroids. Co-stimulatory pathway modulation with Abatacept eventually lead to the normalization of RV function and dilation ten months after initial myocarditis onset.

**Supplementary Information:**

The online version contains supplementary material available at 10.1186/s40959-023-00165-2.

## Background

Immune checkpoint inhibitors mobilize immune system against cancer cells by unmasking T cell tumor immunity and are currently widely used for the treatment of various types of cancers with promising efficacy. However, ICI-induced myocarditis, though uncommon, accounts for high risk of major adverse cardiac events and mortality. The timely and appropriate diagnosis and management of ICI-induced myocarditis is essential for overall patient outcome. We here present a unique, yet challenging case of ICI-induced, refractory and isolated right ventricular myocarditis.

## Case report

**Fig. 1:**
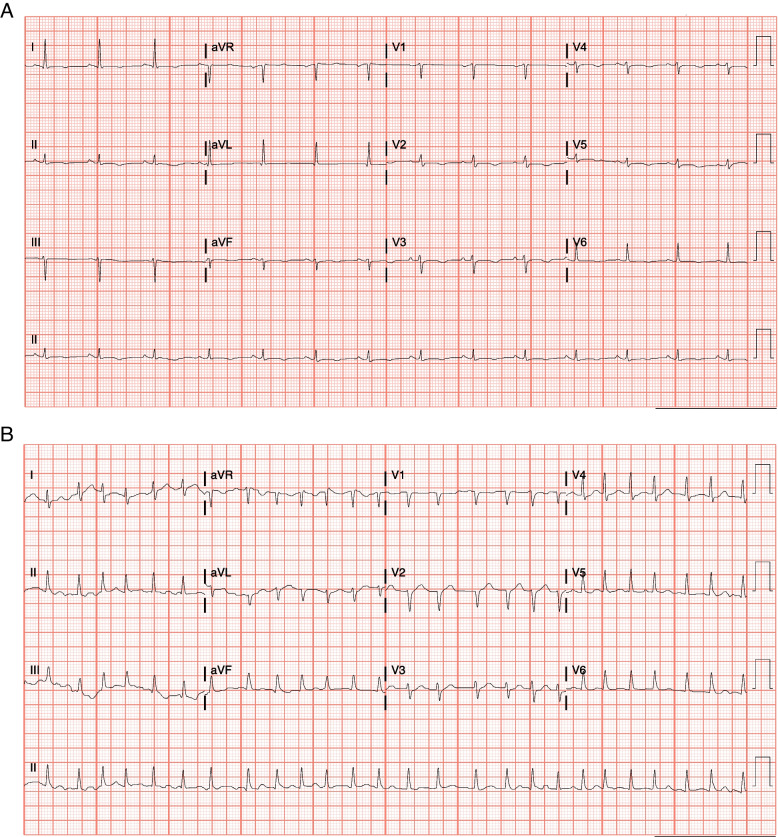
A 12-Lead ECG (**A**) on presentation and telemetry tracing (**B**) during hospitalization showed initial sinus rhythm then evolving into atrial fibrillation with rapid ventricular response

**Fig. 2:**
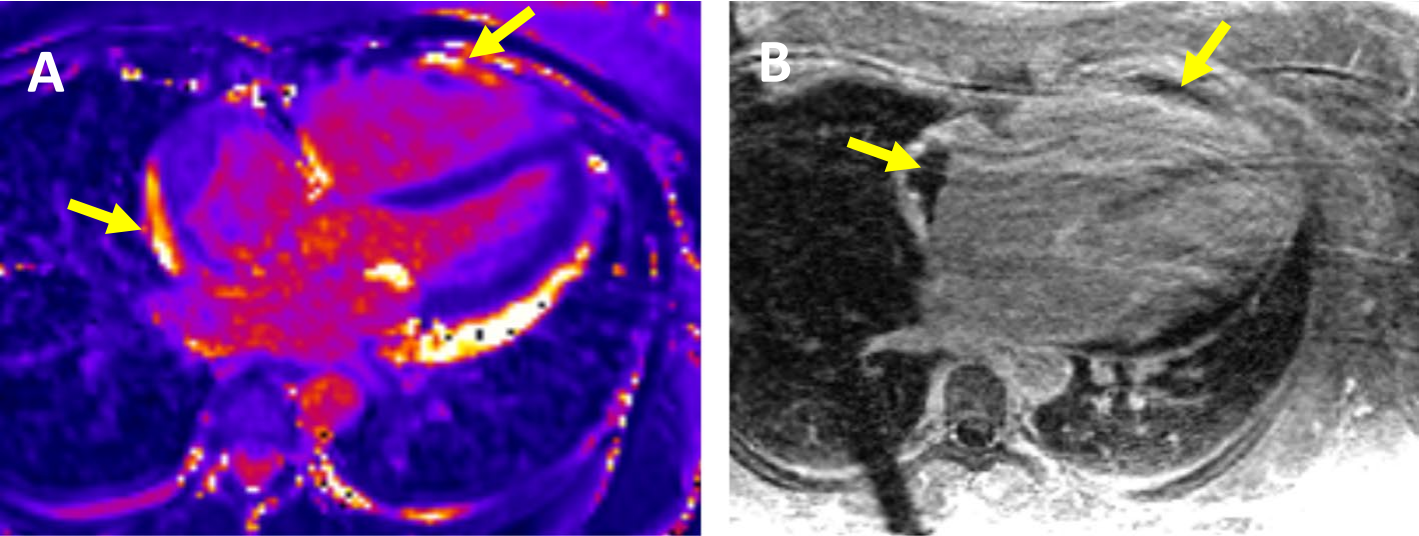
Initial CMR; **A** T2 Map of the myocardium demonstrating increased signal along the right atrium and right ventricle (yellow arrows) suggestive of acute inflammation/edema that does not involve the left ventricle. Increased signal along the left ventricle is related to pericardial effusion and there is no evidence of LV myocardial enhancement or scar. **B** Delayed gadolinium imaging of the heart demonstrating thrombus along areas of inflammation in the right atrium and right ventricle (yellow arrows)

A 32-year-old female with breast cancer presented with newly onset palpitation, chest pain and dyspnea shortly after initiation of Pembrolizumab. The patient was diagnosed with left-sided, triple negative breast cancer 1 year prior, for which she received 4 cycles of carboplatin/paclitaxel ending 7 months prior to the event. She then received 4 cycles of doxorubicin and cyclophosphamide ending 4 months prior, with the final 2 cycles including pembrolizumab as well. Her symptoms started about 6 weeks after initiation of pembrolizumab and gradually worsened. Initial ECG showed sinus rhythm with inferolateral T wave inversion (Fig. 1A) but after admission she developed atrial fibrillation with rapid ventricular response and aberrancy on telemetry (Fig. 1B). Troponin I, CK and B-type natriuretic peptide (BNP) were elevated (Supplemental Fig. 1). Due to concerns of myocardial infarction and acute heart failure, a coronary angiography was therefore performed, which showed normal coronary arteries. Echocardiogram showed left ventricular ejection function (LVEF) 40%, GLS -9.5%, severe RV dysfunction with TAPSE 0.6 cm, and wide-open tricuspid regurgitation. A cardiac magnetic resonance (CMR) was subsequently obtained due to concerns of ICI-induced myocarditis, which revealed diffuse, hyper-enhancement of the RV and RA (with concurrent increased T2 signal) consistent with acute myocarditis primarily involving the right ventricle and right atrium with chamber dilation and severe dysfunction with measured right ventricular ejection fraction of 22% (Fig. 2). The LVEF was 47% on CMR but there was no LV hyper-enhancement or scar noted (Fig. 2), consistent with isolated RV myocarditis. The diagnosis of RV myocarditis was established as directed by current guidelines [1] by exceedingly meeting the clinical diagnosis criteria with Troponin I elevation, plus one major criterion (CMR diagnostic for acute myocarditis as per modified Lake Louise criteria), and additional 2 minor criteria that are not required for diagnosis (clinical syndrome and decline in ventricular function). An endomyocardial biopsy was discussed but felt not indicated after multidisciplinary team discussion and shared decision making with the patient. The following work-ups were also done prior to or during the admission to rule out acute infectious myocarditis: the patient was negative for SARS-CoV-2 by RT-PCR on presentation with two independent testings; She is negative for HIV which was tested before initiating ICI; There was no evidence of any other viral or bacterial infection in her inpatient infectious work-ups, including blood culture and respiratory viral panel, neither did she have any symptoms or signs of recent or active viral or bacterial infection; The patient was last vaccinated for Covid-19 about 8 months prior to the presentation.

The CMR also showed right atrial (RA) dilation with an appendage mural thrombus measured at 4.4 cm × 1.7 cm overlying the areas of RA wall edema. A computed tomography angiogram of the chest showed no evidence of pulmonary embolic disease. To be noted, the pre-chemotherapy echocardiogram 4 months prior showed LVEF of 65%, GLS -18.4%, normal right heart size and function.

In light of the acute RV myocarditis, ICI therapy was stopped immediately and high dose IV steroid therapy was initiated (IV 0.5-g Methylprednisolone daily for three days) followed by oral Prednisone at 60 mg daily tapering over 6 weeks. Therapeutic dose Apixaban was also started for anticoagulation. She was diuresed and her heart failure symptoms resolved with guideline directed medical therapy including beta-blockers, angiotensin II receptor blockers, and loop diuretics.

**Fig. 3:**
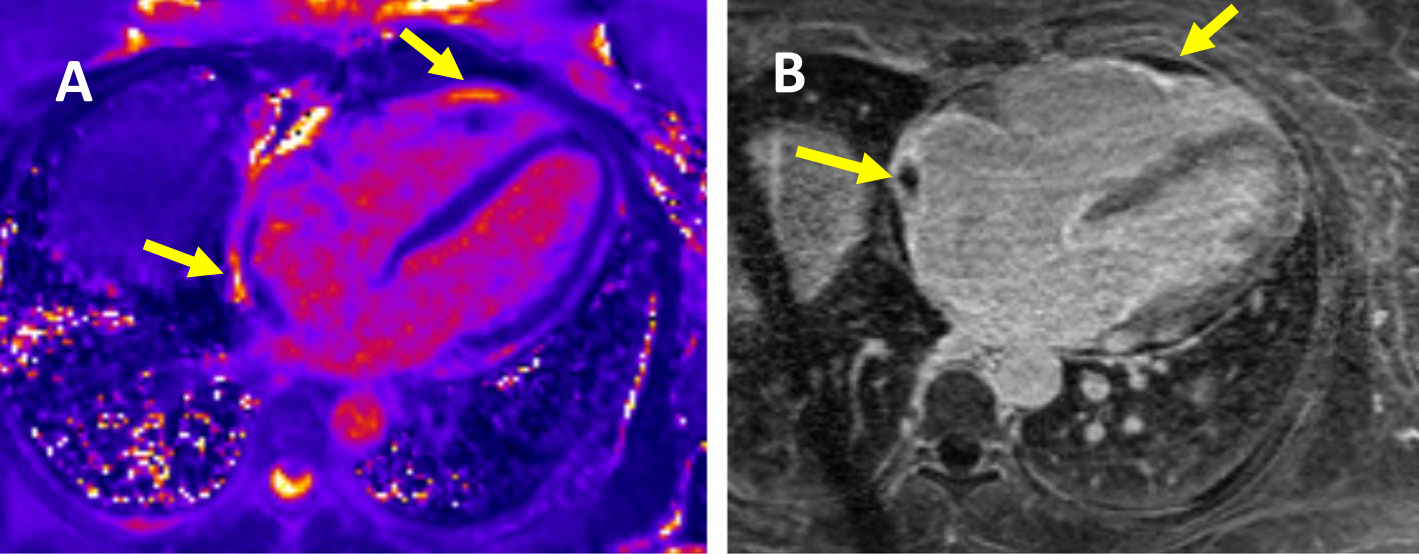
Follow up CMR at 3 months; **A** T2 Map of the myocardium demonstrating increased signal along the right atrium and right ventricle (arrows) suggestive of acute inflammation/edema that does not involve the left ventricle. **B** Delayed gadolinium imaging of the heart demonstrating thrombus along areas of inflammation in the right atrium and right ventricle (yellow arrows)

However, three months after initial presentation, the patient presented to emergency room with decompensated right heart failure and DKA despite compliance with medical therapy. BNP and Troponin I which trended down at 1-month follow-up were found re-elevated during this admission. A repeat cardiac MRI was then obtained which showed recurrent, isolated RV myocardial inflammation/edema without LV involvement and a normalized LVEF of 53% (Fig. 3). This was accompanied by continued severe RV dilation and moderate right atrial dilation with persistent right atrial appendage thrombus despite anticoagulation therapy. High dose oral steroid therapy was started again with slow taper. At 6-month follow up, patient continued to have NY III heart failure symptoms including short of breath and lower extremity edema, and a follow-up CMR continued to show persistent right-sided myocarditis with resolution of right atrial thrombus. Due to refractory RV myocarditis, after risk/benefit discussion and patient consent, she was eventually treated with IV Abatacept at a dose of 10 mg/kg every 2 weeks for total of 3 doses, in addition to the prolonged steroid treatment. Abatacept is an investigative second-line immunosuppression regimen [1, 2], and the dosing we used was based upon prior case report [2] and general dosing recommendation for rheumatoid arthritis treatment. At 9-month follow up, patient remains on chronic low dose Prednisone at 5 mg daily, and she had complete resolution of heart failure symptoms and is off standing diuretics currently. A most recent follow up echocardiogram 9 months after the initial event showed normal LV ejection fraction of 55–60%, GLS -15.8%, and normalized RV cavity size, recovered RV systolic function (TAPSE 1.7 cm) and improved tricuspid regurgitation to trace/mild. Biomarkers including BNP and Troponin I have almost normalized at 10-month follow up (supplemental Fig. 1).

## Discussion and conclusions

Myocarditis refers to active inflammation of cardiac muscle tissue and can present with a wide variety of symptoms including chest pain, dyspnea, and palpitations [3]. Myocarditis can be caused by a multitude of stressors including viral infection, toxic or hypersensitivity drug reactions, giant cell arteritis and sarcoidosis [4]. More recently, immune checkpoint inhibitor have been identified as an important causative agent of myocarditis [5].

A relatively new addition to cancer therapeutics, ICI therapy upregulates the immune response against tumor cells by deactivating inhibitory molecules and receptors that normally dampen active T cell responses [6]. Pembrolizumab, the ICI used by the patient in our case, targets the inhibitory receptor, programmed cell death 1 (PD-1) present on T cells which normally interacts with its ligands PD-L1 and PD-L2 to downregulate the T cell response [6]. However, animal studies have shown that knocking out the PD-1 receptor can lead to uncontrolled systemic autoimmune inflammation, resulting in complications including cardiac dysfunction and development of dilated cardiomyopathy [7]. ICI-induced myocarditis has been a relatively rare complication of cancer immunotherapy with a reported incidence ranging from 0.04% to 1.14% [1, 8–13]. Recent large registry studies and meta-analysis revealed that cardiovascular events during and after ICI treatment are more common than currently appreciated, ranging from 0.3% to ~ 1.8% [14–16]. Despite its relative rarity, ICI-induced myocarditis has been found to be associated with a much higher risk of major adverse cardiovascular events when compared to myocarditis unrelated to ICI therapy [9]. ICI- induced myocarditis generally presents with left ventricular dysfunction as a systematic review found that 67.5% of reported cases with echocardiogram data had an LVEF of less than 50% [10]. However, this case provides an important reminder that LV dysfunction in this setting is neither diagnostic nor suggestive of LV myocarditis, as series CMR had never showed any evidence of LV myocarditis in our case, and LV function promptly recovered which is more suggestive a stress-induced cardiomyopathy process in left ventricle. In addition, literature on right ventricular dysfunction resulting from ICI- induced myocarditis and isolated right-sided myocarditis from ICI therapy is extremely scarce, and report on an isolated, refractory ICI-related RV myocarditis requiring CTLA-4 agonist treatment is non-existent. There is one case report of a patient being treated for pulmonary adenocarcinoma who developed acute right heart failure due to fulminant myocarditis nearly 2 months after initiating treatment with the PD-1 receptor inhibitor Nivolumab [11]. But to the best of our knowledge, our case is the first reported instance of refractory, isolated right-sided myocarditis following ICI treatment, which was successfully treated with Abatacept in addition to long-term steroids. The cause of refractory RV myocarditis despite ICI therapy cessation and immune suppression is unclear and should be a focus of future research in the field, however, it is possible that refractory myocarditis can be minimized with more aggressive initial steroid treatment or earlier use of second-line immunosuppression regimen.

Despite this unusually unilateral presentation, standard therapy for ICI- induced myocarditis with high-dose steroid therapy can be considered a reasonable treatment option in this instance. The European Alliance of Associations for Rheumatology (EULAR) and European Society of Cardiology recently released guidelines recommending high-dose systemic glucocorticoids (1–2 mg/kg/day) as first-line treatment for ICI-induced myocarditis [1, 17]. Additionally, conventional synthetic disease-modifying antirheumatic drugs (csDMARDs) such as mycophenolate mofetil and methotrexate may be considered in glucocorticoid-refractory cases despite current lack of extensive evidence given the severity of disease that can occur [1, 17]. CTLA-4 agonist, Abatacept, has been mentioned in current guidelines and case studies as a promising, investigative second-line immunosuppression regimen [1, 2], in the case, refractory ICI-induced RV myocarditis was successfully treated with combination therapy of Abatacept and long-term steroids, suggestive a mortality/morbidity benefit from CTLA-4 modulation in this patient population, therefore a randomized clinical trial on this very question is of imminent need.

In conclusions, we describe the first case of isolated, refractory ICI-induced right ventricular myocarditis leading to recurrent right ventricular failure, despite ICI therapy cessation and immune suppression by repeated high dose steroids. Co-stimulatory pathway modulation with Abatacept eventually lead to the normalization of RV function and dilation nine months after initial myocarditis onset and biomarkers normalized at 10-month follow-up, highly suggestive a potentially effective treatment option for ICI-induced refractory RV myocarditis.

## Supplementary Information

**Additional file 1: Supplemental Figure 1.** Trending of CK, BNP and Troponin I biomarkers during treatment course.

## Data Availability

N/A.

## Abbreviations

BNP: B-type natriuretic peptide
CMR: Cardiac magnetic resonance imaging
csDMARD: Conventional synthetic disease-modifying antirheumatic drugs
DKA: Diabetic ketoacidosis
EULAR: European Alliance of Associations for Rheumatology
ICI: Immune checkpoint inhibitor
LV: Left ventricle
LVEF: Left ventricular ejection function
PA: Pulmonary artery
PD-1: Programmed cell death 1
RA: Right atrium
RV: Right ventricle
TTE: Transthoracic echocardiogram
