# Supplementary figures and images for: Refractory right ventricular myocarditis induced by immune checkpoint inhibitor despite therapy cessation and immune suppression
Source: Cardiooncology. 2023 Mar 20;9:15. doi: 10.1186/s40959-023-00165-2 (PMC10026228; doi:10.1186/s40959-023-00165-2)

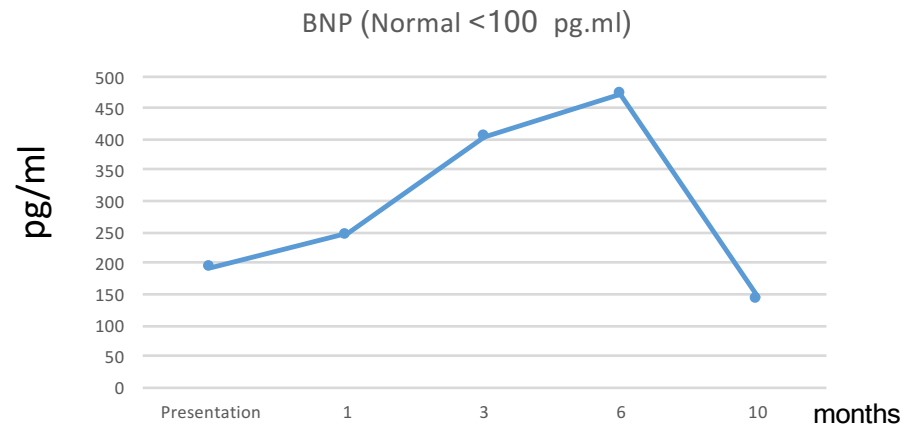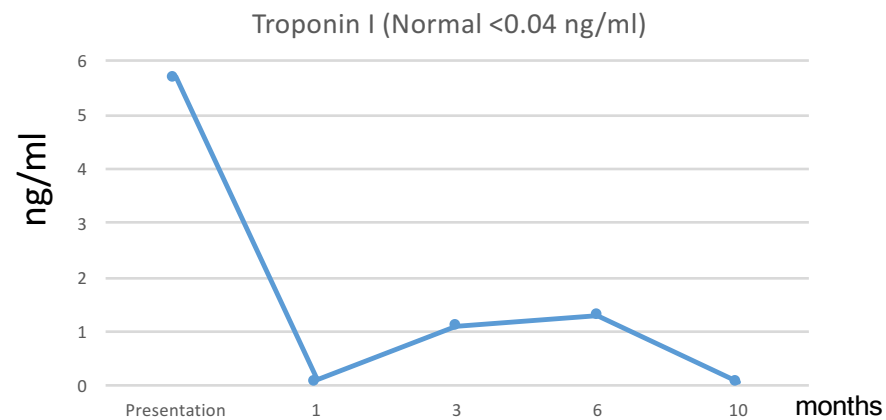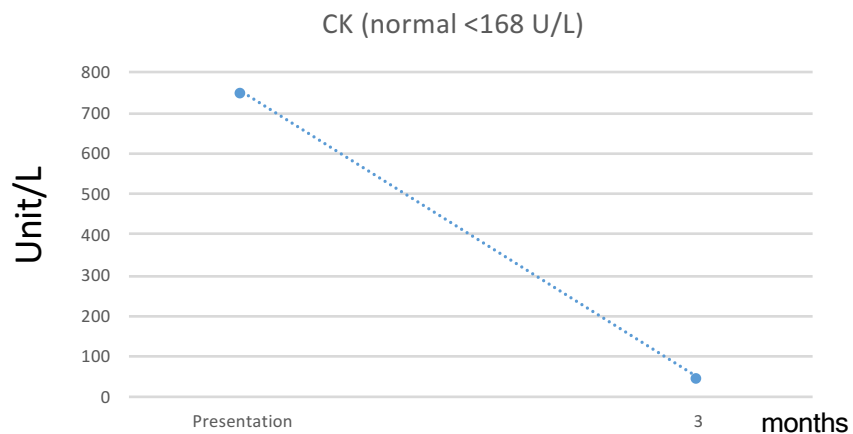

Supplement: Supplementary file 1 — Additional file 1: Supplemental Figure 1. Trending of CK, BNP and Troponin I biomarkers during treatment course. [file 40959_2023_165_MOESM1_ESM.pdf]
